# Chromosomal instability as an architect of the cancer stemness landscape

**DOI:** 10.3389/fcell.2024.1450614

**Published:** 2024-09-13

**Authors:** Shahnawaz A. Baba, Aran Zakeri, Jay S. Desgrosellier

**Affiliations:** ^1^ Department of Pathology, University of California, San Diego, La Jolla, CA, United States; ^2^ Moores Cancer Center, University of California, San Diego, La Jolla, CA, United States

**Keywords:** chromosomal instability, cancer stem cell, stress tolerance, inflammation, breast cancer, metastasis

## Abstract

Despite a critical role for tumor-initiating cancer stem cells (CSCs) in breast cancer progression, major questions remain about the properties and signaling pathways essential for their function. Recent discoveries highlighting mechanisms of CSC-resistance to the stress caused by chromosomal instability (CIN) may provide valuable new insight into the underlying forces driving stemness properties. While stress tolerance is a well-known attribute of CSCs, CIN-induced stress is distinctive since levels appear to increase during tumor initiation and metastasis. These dynamic changes in CIN levels may serve as a barrier constraining the effects of non-CSCs and shaping the stemness landscape during the early stages of disease progression. In contrast to most other stresses, CIN can also paradoxically activate pro-tumorigenic antiviral signaling. Though seemingly contradictory, this may indicate that mechanisms of CIN tolerance and pro-tumorigenic inflammatory signaling closely collaborate to define the CSC state. Together, these unique features may form the basis for a critical relationship between CIN and stemness properties.

## Introduction

**FIGURE 1:**
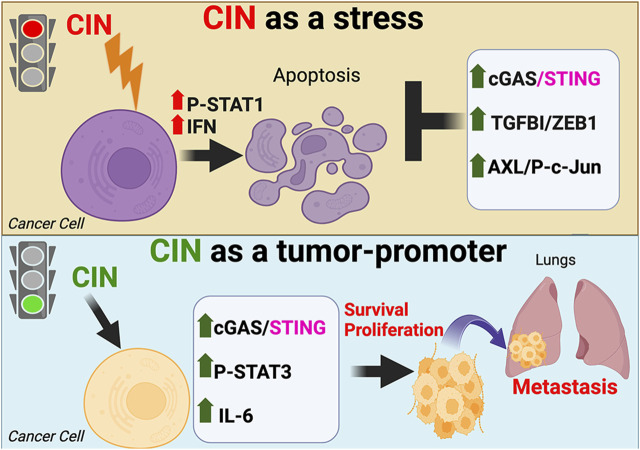
The dual effects of chromosomal instability (CIN) on cancer cells. CIN can paradoxically act as a lethal stress limiting the ability of tumor cells to transform or progress (top), or initiate pro-metastatic antiviral signaling that enhances cell survival and proliferation (bottom).

CIN is a widely appreciated hallmark of cancer (Hanahan and Weinberg, 2011). Defined by continuous chromosomal missegregation, CIN is distinct from aneuploidy, which represents a state of abnormal chromosome number (Holland and Cleveland, 2009). The combined effects of CIN and aneuploidy play a well-described role in tumor evolution by promoting karyotypic heterogeneity (Bakhoum and Compton, 2012; Gronroos and Lopez-Garcia, 2018) and is thoroughly reviewed elsewhere (Chen et al., 2024; Hosea et al., 2024). By altering gene dosage, CIN can promote amplification of new oncogenes and deletion of tumor suppressors that aid cancer evolution and resistance to therapy (Benner et al., 1991; Turner et al., 2017; Albertson, 2006; Lukow et al., 2021). However, inherent to all of these effects is the ability of cancer cells to first tolerate the stress induced by CIN. Thus, the ability to resist this stress must be an important feature of aggressive breast cancer cells. To distinguish the effects of CIN versus aneuploidy on breast cancer progression, approaches now exist to specifically measure CIN by quantifying chromosomal missegregations during anaphase (Bakhoum and Compton, 2012), or assessing enrichment for CIN gene signatures (Bakhoum et al., 2018; Carter et al., 2006). Studies using these approaches have shown that CIN levels are higher in metastatic lesions compared to matched primary disease, suggesting that this stress is induced during metastasis (Bakhoum et al., 2018). These high CIN levels may pose a significant barrier preventing metastatic outgrowth and selecting for the most CIN-resistant cells. Furthermore, distinguishing CIN’s effects from those due to aneuploidy revealed that CIN mediates unique biological responses. These include roles in tumor initiation/metastasis (Bakhoum et al., 2018), resistance to stress (Hong et al., 2022), and activation of pro-tumorigenic signaling pathways (Bakhoum et al., 2018; Hong et al., 2022), placing CIN at the intersection between stress tolerance and CSC properties (Figure 1). Together, this makes CIN’s potential function in defining cancer stemness an appealing target for further investigation.

### Resistance to CIN-induced stress as a hallmark of CSCs

Although CIN is a hallmark of cancer (Hanahan and Weinberg, 2011), little is known about its relationship with stemness. Breast cancer CSCs bearing similarities to adult mammary stem cells (MaSCs) are important contributors to metastasis and disease progression (Al-Hajj et al., 2003; Lim et al., 2009; Prat and Perou, 2011; Malanchi et al., 2011). Stress tolerance is a key property associated with these CSCs, including resistance to exogenous stresses such as chemotherapy (Lytle et al., 2018) or hypoxia (Abd et al., 2023) as well as endogenous stresses caused by metabolic activity (Lee et al., 2015) or DNA damage (McGrail et al., 2018; Vitale et al., 2017). In addition to its role in tumor evolution, CIN is a significant stress that reduces cancer cell fitness (Gronroos and Lopez-Garcia, 2018). As a tumor-specific stress, all cancer cells exhibit some level of CIN resistance. In fact, CIN’s effects as a stress appear to outweigh any advantage posed by the increased karyotypic heterogeneity, as enhancing CIN levels negatively impacted glioblastoma CSCs (Godek et al., 2016). This emphasizes the importance of CIN tolerance in shaping stemness properties, as high CIN levels can tip even CSCs toward cell death. Consistent with this idea, recent studies have identified CIN as an endogenous stress more effectively tolerated by CSCs compared to non-stem cell types (Hong et al., 2022; Morel et al., 2017; Sun et al., 2022; Baba et al., 2023). While the acquisition of CIN is a normal part of aging, these cells are efficiently targeted for death and removal, and thus CIN is usually not found in healthy tissues (Barroso-Vilares et al., 2020; Chen et al., 2023). For this reason, discovery that normal mammary stem cells were better equipped to resist this stress during oncogenic transformation was somewhat unexpected (Morel et al., 2017). This may indicate that CIN tolerance is a conserved property of both normal stem cells and CSCs. In this same vein, it appears that the response to CIN may vary among different cell types, even in the same tissue, since altering the levels of CIN in mice predisposed to intestinal cancer showed striking differences in adenoma formation in distinct regions of the intestine (Hoevenaar et al., 2020). Thus, despite its role in cancer evolution, CIN-induced stress may be of more vital importance in shaping the heterogeneous tumor landscape, with CSCs characterized by enhanced resistance.

### ZEB1 as a mechanism of CIN resistance

**FIGURE 2:**
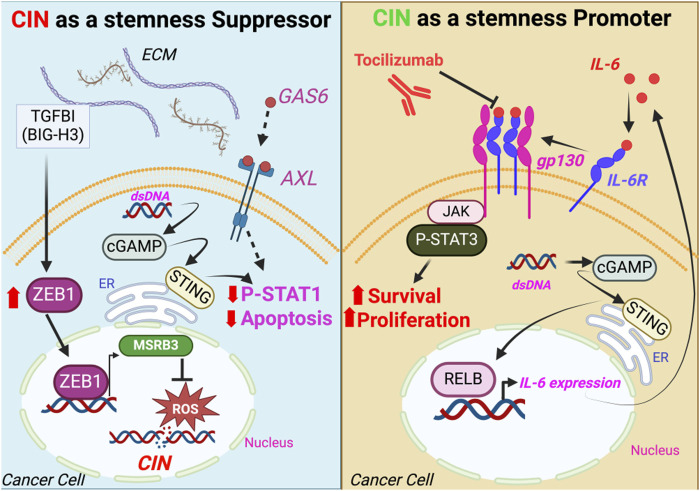
Signaling pathways mediating the CSC response to CIN. CSC resistance to the stress caused by CIN occurs through preventing DNA damage or inactivating STAT1-mediated cell death (left panel), while STING-induced cytokines such as IL-6 drive cell survival and proliferation via STAT3 activation (right panel). ECM, extracellular matrix; dsDNA, double-stranded DNA; ER, endoplasmic reticulum.

CIN is a universal feature of all cancer cells, however, recent studies have discovered enhanced CIN tolerance associated with CSCs (Hong et al., 2022; Morel et al., 2017; Sun et al., 2022; Baba et al., 2023) as well as normal MaSCs exposed to oncogenes (Morel et al., 2017). These cells possessed unique mechanisms underlying this behavior that provided a survival advantage over other non-stem cell types (Figure 2). By performing unbiased analysis of critical genes expressed by CSCs in an activated signaling state, *ZEB1* and *TGFBI* (BIG-H3) were identified as critical for CIN tolerance (Sun et al., 2022). Further characterization revealed that these two independently identified genes were related as part of a TGFBI (BIG-H3)-ZEB1 signaling module (Sun et al., 2022), with TGFBI (BIG-H3) stimulating ZEB1 mRNA and protein levels (Sun et al., 2022). This presumably occurs through activation of Transforming Growth Factor-beta signaling, although the precise mechanism has yet to be elucidated. These findings were remarkably consistent with those from normal human MaSCs, where *ZEB1* gene expression was identified as critical for resistance against CIN-induced stress caused by oncogenic HRAS (Morel et al., 2017). Further studies showed that these effects were due to ZEB1-mediated transcription of the methionine sulfoxide reductase *MSRB3*, which prevented DNA damage induced by reactive oxygen species (Morel et al., 2017). The stress caused by CIN served as a significant impediment to oncogenic transformation of normal human breast epithelial cells, while MaSCs expressing ZEB1-MSRB3 were more likely to be transformed since they could tolerate CIN (Morel et al., 2017). Similarly, TGFBI (BIG-H3) was also previously shown to mediate CIN resistance, as *TGFBI* knockout mice displayed enhanced levels of chromosomal aberrations and micronuclei (Zhang et al., 2009). Additionally, deletion of ZEB1 or TGFBI (BIG-H3) in breast cancer cells enhanced sensitivity to PARP inhibitors (Sun et al., 2022), suggesting that CIN resistance mechanisms may represent potential therapeutic vulnerabilities. Together, these findings highlight ZEB1 as a conserved mechanism of CIN tolerance present in both normal stem cells and CSCs.

### c-Jun/AXL as CSC-specific enhancers of CIN tolerance

Additional mechanisms involved in CSC resistance to CIN include a new role for the receptor tyrosine kinase AXL. Recent studies discovered *AXL* as a gene induced by activation of c-Jun/JNK stress signaling in CSCs, allowing these cells to tolerate chronic endogenous stress caused by CIN (Sun et al., 2022). This is consistent with previous roles described for AXL inhibition in blocking the DNA damage response and increasing sensitivity to PARP inhibitors (Ramkumar et al., 2021), as well as for JNK activation as necessary for CIN tolerance (Wong et al., 2014). In fact, both *JUN* and *AXL* were found to be enriched in CSCs (Sun et al., 2022) as well as cells with high-CIN (Bakhoum et al., 2018), suggesting that their expression might be part of the CIN response. This highlights a significant role for c-Jun/AXL signaling in mediating CIN tolerance in CSCs. These effects may be due to AXL’s ability to suppress STAT1-mediated cell death. STAT1 is required for CIN-induced cell death (Hong et al., 2022), which is deficient in aggressive cancer cells due to chronic inactivation of this pathway (Li et al., 2023). In other cell types, AXL has been shown to block STAT1-dependent responses (Rothlin et al., 2007), thus it is tempting to speculate it may similarly function to block the STAT1 tumor suppressor in CSCs, enhancing CIN tolerance. Future studies will explore how this new role for AXL may be related to its previously characterized functions in innate immunity (Bottai et al., 2016), including potential impacts on the STING/IL-6 pathway downstream of CIN (Bakhoum et al., 2018; Hong et al., 2022). Such findings may suggest similarities between AXL’s role in regenerating the epithelium after a viral infection (Fujino et al., 2019) and its effects downstream of CIN in cancer, such that AXL may maintain CSCs in a persistent activated state that underlies their metastatic potential. Despite similar impacts on CIN resistance, the c-Jun/AXL and TGFBI (BIG-H3)-ZEB1 pathways do not appear to crosstalk and instead may act in parallel, since only TGFBI (BIG-H3)-ZEB1 directly affected CIN levels. Taken together, these findings highlight important mechanisms for properly regulating the response to CIN-induced stress in CSCs. This raises the possibility that CIN tolerance pathways could be responsible for the aggressive nature of CSCs, underlying their role as key initiators of metastasis (Lawson et al., 2015).

### A potential CSC role for CIN-induced antiviral responses

While CSCs are capable of resisting many different forms of stress, CIN is unique, as it can activate pro-tumorigenic signaling pathways (Figure 2). Despite being a significant endogenous stress that limits transformation potential (Morel et al., 2017), studies using specific KIF2C constructs to modify CIN levels (Moore et al., 2005; Bakhoum et al., 2009) have shown that CIN can paradoxically enhance tumor progression and metastasis by inducing inflammatory signaling (Bakhoum et al., 2018; Hong et al., 2022; Li et al., 2023). These distinct responses to CIN act as two sides of the same coin, with CIN resistance representing an important prerequisite for any pro-tumor responses. Enhanced levels of CIN resulted in micronuclei, which ruptured to produce dsDNA in the cytoplasm (Bakhoum et al., 2018; Hong et al., 2022; Mackenzie et al., 2017). This was then sensed by the cGAS/STING pathway, initiating a chronic antiviral signaling response (Bakhoum et al., 2018; Hong et al., 2022). Of note, this did not activate the canonical downstream interferon/NFKB cell death, but instead initiated a tumor pro-survival and pro-proliferation response (Bakhoum et al., 2018; Hong et al., 2022), highlighting a crucial distinction between the CIN response and signaling due to a viral infection. While activation of cGAS/STING normally induces cell death due to interferon/TLR/STAT1, CIN-induced STING signaling resulted in enhanced survival and proliferation due to non-canonical NFKB (RelB) (Bakhoum et al., 2018) as well as IL-6/JAK2/STAT3 signaling (Hong et al., 2022). This may be due to inactivation of the STAT1 cell death response in aggressive cancer cells, leaving only the pro-tumorigenic response intact. While both responses are downstream of cGAS/STING, it appears likely that unique genes or pathways specific to aggressive cells may tip the balance toward the pro-tumorigenic response. Deciphering the specific mechanisms that provide this context in CSCs will likely furnish significant insight into what makes these cells so malignant. However, it is an unanswered question as to whether the pro-tumor antiviral signaling downstream of cGAS/STING acts in concert with CIN tolerance pathways or represents a parallel signaling module. Studies suggest that the chronic activation of STING may itself lead to tolerance (Hong et al., 2022). In summary, recent findings highlight an important role for CIN not only as a stress CSCs must tolerate, but as a potential cause of aggressive breast cancer cell properties.

An important caveat of these studies is that all studies to date were performed in stem-like claudin-low breast cancer cell lines. Thus, it remains to be determined whether luminal or basal-like breast cancer cells will respond in the same manner. This is particularly important since STING activation appeared to be uncoupled from CIN in ER+ patient breast cancers. Tumors with activated STING were associated with a robust immune response and overall positive prognosis, whereas high CIN levels were noted in ER+ cancers with low STING activation and poor prognosis (Parkes et al., 2021). Interestingly, there was no correlation between STING signaling and CIN in ER- patient cancers in this study. This may be partly due to the fact that only moderate CIN levels indicated a poor prognosis in Triple-Negative disease, whereas cancers with high levels of CIN had a better outcome (Jamal-Hanjani et al., 2015). More information is needed about the context in which the CIN/STING signaling axis occurs and what if any cell type-dependent co-factors are necessary to achieve this response.

### Cooperation between CIN’s two roles in shaping CSC behavior

Although CIN’s role as both a cancer-specific stress and a tumor-promoter may appear contradictory, their cooperation may be critical for CSC properties. The ability of CSCs to tolerate high CIN levels would not only allow them to survive, but may also function as a prerequisite for any pro-tumor signaling response. By viewing these effects together, we may gain a more complete perspective of CIN’s role in cancer progression. Furthermore, since studies suggest that CIN levels may be dynamic during tumor initiation and metastasis, this vantage point may allow us to better identify the most critical stages and cell types affected by CIN. During metastasis, CIN levels have been observed to increase dramatically compared to matched primary tumors (Bakhoum et al., 2018). Similarly, our lab noted higher amounts of CIN in low-burden versus high-burden tumors from CSCs, suggesting that levels increase during the initial phases of tumor initiation and decrease as tumors become more established (Baba et al., 2023). Importantly, basal levels were similar between CSC and non-stem cell types prior to injecting into mice (Baba et al., 2023), consistent with an increase in CIN specific to early stage tumors. Since non-CSCs displayed reduced tumor-initiating ability, this may indicate that they are less able to tolerate this stress, with increased CIN serving as a critical barrier to the initiation of new tumors. Additionally, this suggests that CSCs possess the ability to tolerate high levels of CIN, underlying their role in initiating new tumors during the early stages of metastasis (Lawson et al., 2015). The differing effects of CIN in CSCs versus non-stem cells is consistent with studies showing that the oncogenic response to CIN varies in distinct tissues and cell types (Morel et al., 2017; Hoevenaar et al., 2020). Overall, these findings suggest that CIN may be induced during early tumor initiation, favoring a role for CSCs, whereas CIN appears to diminish as tumors grow and differentiate, allowing other cell types to become ascendant. Furthermore, the higher CIN levels tolerated by CSCs could also provide an advantage by inducing pro-tumor STING signaling, aiding their ability to survive and grow. Thus, this model of dynamic changes in CIN during tumor progression highlights how both pro-tumor and stress tolerance pathways may cooperate to enhance stemness and initiate new cancers.

### CIN’s contribution to CSC immune evasion

In addition to affecting cancer cell behavior directly, CIN also impacts the tumor microenvironment (TME), primarily by regulating the recruitment and activation of immune cells. High levels of CIN appear to create an immunosuppressive TME due to reduced lymphocyte infiltration and defects in antigen presentation, causing decreased responsiveness to immunotherapies (Taylor et al., 2018; Davoli et al., 2017; Tripathi et al., 2019). This is somewhat unexpected since higher levels of CIN would be predicted to enhance the levels of neoantigens in cancer cells due to higher mutational burden. At least some of the effects of CIN on cancer immune evasion appear to involve cGAS/STING signaling (Li et al., 2023), which re-wires the immune landscape towards one that is pro-metastatic (Li et al., 2023). However, this too may be paradoxical, as CIN also appears to enhance susceptibility to anti-tumor immune responses by activating macrophages (Hayes et al., 2024). The role of CSCs in mediating CIN’s effects on the TME are still very much unclear. Given their scarcity, it seems unlikely that CSCs themselves produce an outsized response in the TME. Rather, it seems more probable that CIN contributes toward making CSCs particularly well-suited to immune evasion. Consistent with this possibility, CSCs evade the immune system by expressing high levels of PD-L1 and abrogating the anti-tumor effects of interferon signaling (Hsu et al., 2018), similar to the immunosuppression caused by CIN in cancer. This is may be to blame for the reported negative correlations between stemness and cancer immunity (Miranda et al., 2019). Thus, CIN may aid CSC immune evasion, enhancing their metastatic potential and leading to resistance to immune checkpoint blockade. CIN’s effects on immune regulation likely stem from increased cytokine secretion by cancer cells. Interleukin-6 (IL-6) has already been characterized as one such secreted signaling factor increased by CIN/STING signaling (Hong et al., 2022). While IL-6 has been shown to induce paracrine signaling in cancer cells resulting in increased proliferation and survival (Hong et al., 2022), it may also influence the immune microenvironment. Overall, there is still much to learn about the specific cancer cell-dependent and independent effects induced by CIN, especially with regard to CSCs and other cell type-dependent differences. Future studies will be needed to determine the role of CIN in mediating CSC immune evasion and prioritize the various pro-tumor cytokine signaling networks for their potential use as therapeutic targets.

## Discussion

Taken together, these studies highlight CIN’s important contribution to promoting stemness features. Since CSCs have a disproportionate effect on cancer progression, there is a critical need for more effort in the field geared toward addressing the role that stresses, such as CIN, may play in modulating, and even driving CSC behavior. Toward this goal, there remain several critical unresolved questions that will serve as the basis for future studies. These include addressing how aggressive cancer cells, such as CSCs, can uniquely harness CIN to induce pro-tumorigenic cGAS/STING signaling relative to other cells. Recent studies indicate that CIN levels are similar in stem and non-stem cell types (Baba et al., 2023), suggesting that it is not sufficient for stemness. This may provide a rationale for future work to define factors that specify the unique CSC response to CIN. Central to this effort will be the elucidation of the sentinel antiviral response genes that underly any cell type or subtype-dependent differences in STING activity. This may include unique differences in the downstream genes expressed due to STING activation in CSCs. Identifying crucial CIN tolerance pathways is a key next step in these studies. This will address important questions regarding why aggressive cells lose sensitivity to interferon-induced STAT1-mediated cell death. While more tolerant of CIN, CSCs still have the capacity to undergo apoptosis due to elevated CIN levels (Godek et al., 2016), indicating that re-activating the pro-death response may be an attractive therapeutic approach. By targeting CIN tolerance pathways, this could re-sensitize aggressive cells to interferon-induced cell death, using the cell’s own CIN-induced STING response against it. This area of research may represent a promising new avenue with regard to potential new breast cancer therapies. By defining CIN’s role in stemness, and identifying the mechanisms that confer tolerance against this chronic insult, studies in this area may result in a major advance in our understanding of breast cancer stemness and shed light on the underlying cause of metastasis. This could potentially characterize aggressive cancers as less like a wound, and more similar to a chronic viral infection.
